# 

**Published:** 1881-10

**Authors:** 


### CONTENTS:

Proceedings of the Southern Dental Association, conjointly with the North Carolina State Dental Association.—Thirteenth Annual Meeting.

